# Pathogenic piscine intestinal coccidia infection alters gut microbiome in juvenile Asian seabass (*Lates calcarifer*)

**DOI:** 10.1186/s12917-025-05168-y

**Published:** 2025-12-18

**Authors:** Songphon Buddhasiri, Sasibha Jantrakajorn, Chayanis Daochai, Narissara Keawchana, Peerapon Sornying, Parameth Thiennimitr, Watcharapol Suyapoh

**Affiliations:** 1 Veterinary Public Health and Food Safety Centre for Asia Pacific, Faculty of Veterinary Medicine, Chiang Mai University, Chiang Mai, 50100 Thailand; 2 Faculty of Veterinary Medicine, Chiang Mai University, Chiang Mai, 50100 Thailand; 3 Faculty of Veterinary Science, Prince of Songkla University, Songkhla, 90110 Thailand; 4 Department of Microbiology, Faculty of Medicine, Chiang Mai University, Chiang Mai, 50100 Thailand

**Keywords:** Dysbiosis, Gut microbiota, Microbiome, Pathogenesis, Pathogenic bacteria, Piscine intestinal coccidia, *Lates calcarifer*

## Abstract

**Background:**

Piscine intestinal coccidiosis has been associated with increased susceptibility to secondary bacterial infections, potentially through disruption of the gut microbiota. While many gut bacteria are commensal, infection-related alterations in microbial composition may favor the proliferation of opportunistic taxa. However, the specific effects of intestinal coccidial infections on the gut microbiome of fish remain insufficiently described.

**Results:**

This study examined the influence of intestinal coccidial infection on gut microbiota composition and the relative abundance of potentially pathogenic bacteria in juvenile Asian seabass (*Lates calcarifer*). Intestinal samples from seven coccidia-infected and five uninfected fish were analyzed using 16S rRNA gene sequencing. Infected fish demonstrated greater microbial diversity and marked compositional shifts across multiple taxonomic levels. Relative abundances of genera with reported pathogenic potential, such as *Clostridium*, *Lactococcus*, and *Kurthia*, as well as zoonotic bacteria including *Helicobacter* and *Escherichia-Shigella*, were elevated in the infected group. Conversely, uninfected fish harbored higher levels of genera such as *Bacillus*, *Vibrio*, *Acinetobacter*, and *Streptococcus*, which are commonly associated with environmental niches and opportunistic colonization. Coccidial infection was also linked to microbial imbalance, reflected in reduced Proteobacteria-to-Bacteroidota (P:B) and Proteobacteria-to-Firmicutes (P:F) ratios, alongside histopathological evidence of intestinal inflammation.

**Conclusions:**

Intestinal coccidiosis in juvenile Asian seabass is associated with open niche gut characterized by altered microbial diversity and increased abundance of bacterial genera with pathogenic potential. These findings suggest that coccidial infection may contribute to an elevated risk of secondary infections, underscoring the relevance of gut microbiome monitoring in fish health management. Further studies are warranted to clarify host–parasite–microbiome interactions and their implications for aquaculture health.

**Supplementary Information:**

The online version contains supplementary material available at 10.1186/s12917-025-05168-y.

## Background

Piscine intestinal coccidia infection is a significant parasitic health issue for juvenile Asian seabass (*Lates calcarifer*) in Southeast Asia, including Thailand and Vietnam, where over 90% of the juvenile fish are affected [1, 2]. The common coccidia reported in Asian seabass belong to the genera *Eimeria* spp. and *Cryptosporidium* spp. [3]. It is suspected that these parasites are transmitted to fish through invertebrate food [4]. Once ingested, the sporocysts excyst and invade the fish's intestinal epithelium, completing their life cycle [5]. This infection causes damage through both mechanical and molecular effects, leading to various intestinal abnormalities, such as enteritis, mucosal denudation, and intestinal necrosis [1, 6]. Clinical manifestations of coccidia infections in fish can range from asymptomatic cases to severe symptoms and increased mortality [7]. The severity of these symptoms depends on various factors such as the fish species, age, size, culture conditions, the specific coccidia species, and the intensity of the parasitic infection [3, 8–10]. Notably, experimental studies suggested that infections with piscine intestinal coccidia may heighten fish susceptibility to secondary bacterial infections caused by *Pseudomonas* spp*., Aeromonas* spp*.,* and *Exiguobacterium* spp*.* [11]. While most of these bacteria are part of the normal gut flora in fish, they can also act as opportunistic pathogens under certain conditions.

The gut microbiota is a diverse community of microorganisms that plays key roles in immune response, digestion, and metabolism in teleost fish [12–15]. In higher vertebrates, alterations in microbial flora caused by coccidia can lead to open niche gut [16]. This microbial alteration in the gut microbiome is linked to an increase in pathogenic microbes, which can lead to the translocation of these overgrowing bacteria into the bloodstream, contributing to systemic organ infections, damage, and dysfunction [17]. However, the potential impact of piscine intestinal coccidia on changes in the host's microbiome remains relatively unexplored. This influence may facilitate the proliferation of pathogenic bacteria, but there has been limited research conducted in this area.

To gain a deeper understanding of how piscine intestinal coccidia affects the intestinal microbiome in juvenile Asian seabass, we investigated changes in gut microbiota of fish naturally infected with coccidia compared to uninfected fish. We utilized genomic sequencing technologies to analyze the intestinal contents and tissues of both infected and control fish. This approach allowed us to explore the impact of coccidia infection on the intestinal microbiota and the proliferation of harmful gut microbes. The objective of this study is to provide insights into how piscine intestinal coccidia infection alters the intestinal microbiota and supports the growth of bacterial pathogens in juvenile Asian seabass.

## Materials and methods

### Experimental design and sample collection

Thirteen juvenile Asian seabass (*Lates calcarifer*) were obtained in December 2024 from a privately owned aquaculture facility in Satun Province, Thailand. The fish were reared in rectangular sea cages with a volume of 50 m^3^, each stocked at a density of approximately 1,000 fingerlings. The cages were deployed in the open sea, where water quality parameters remained stable, with salinity at 35 ppt, temperature at 25 °C, and pH at 8.2. Fingerlings measuring 2.5–5.0 cm in length were initially fed ground trash fish at 8–10% of body weight per day, divided into four to five feedings. As the fish grew, their diet was gradually transitioned to finely chopped trash fish. For transport, fish were collected live and placed in aerated plastic containers before being transferred to the Department of Veterinary Sciences, Faculty of Veterinary Science, Prince of Songkla University. Upon arrival, they were acclimated under controlled temperature and photoperiod conditions and subjected to a 12-h fasting period before sampling. All fish underwent a comprehensive health assessment, including measurement of body length and weight, clinical and postmortem observations, and evaluation for external parasites. Euthanasia was then performed by immersion in a 100 mg/L solution of Aquanes® (Better Pharma, Bangkok, Thailand), with eugenol as the active compound. Fish were confirmed to be fully anesthetized and unresponsive, and were maintained in the solution for at least 20 min following cessation of opercular movement to ensure complete euthanasia. Intestinal samples were subsequently collected from all thirteen fish, comprising five uninfected individuals and eight infected with coccidia. Each intestine was divided into anterior and posterior segments. The anterior sections were fixed in 10% neutral buffered formalin for 24 h for histopathological examination, whereas the posterior sections were rinsed with sterile phosphate-buffered saline (PBS), transferred to cryovials, snap-frozen in liquid nitrogen, and stored at –80 °C for subsequent molecular and biochemical analyses.

### Clinical, postmortem, and external parasitological examinations

Prior to euthanasia, all fish were carefully examined for clinical abnormalities, including swimming behavior, respiratory patterns, body condition, body surface, and external organs. Euthanasia was then performed by immersion in a 100 mg/L solution of Aquanes® (Better Pharma, Bangkok, Thailand), containing eugenol as the active ingredient. Fish were maintained in the solution for at least 20 min after cessation of opercular movement to ensure complete euthanasia, in accordance with the protocol of Suyapoh et al. [18]. After euthanasia, each fish was measured for total body length and body weight, followed by a systematic postmortem examination. Gross pathological changes in internal organs were observed.

External parasitological assessment was conducted by inspecting the skin, fins, and opercular cavities for external parasites. In addition, gill filaments were dissected, and wet-mount preparations were examined under a light microscope at 40–400 × magnification. The number of parasites per infested host was recorded to determine infestation intensity, which was classified according to Bush et al. [19]. The degree of ectoparasite infestation per 100 × microscopic field (average of 10 fields) was graded as follows: no parasites detected (–); light ( +), with one parasite per field; moderate (+ +), with 2–5 parasites per field; and heavy (+ + +), with more than five parasites per field [20].

### Detection and assessment of coccidia

Coccidia detection was carried out by histological examination of the small intestine using a Nikon advanced upright microscope equipped with a video capture digital camera (ECLIPSE Ni-U; Nikon, Tokyo, Japan). Parasites were evaluated at three distinct epithelial locations: epicellular, intracytoplasmic, and extracellular (including subepithelial and submucosal positions), and counts were performed following the protocol described by Suyapoh et al. [1]. Morphological identification of developmental stages was conducted according to criteria outlined in previous studies [5, 21] and confirmed by a parasitologist to ensure diagnostic accuracy. The total number of parasites per fish was then used to calculate infection intensity, which served as the primary metric for assessing the severity of coccidial infection.

### DNA extraction and 16S rRNA gene sequencing

Microbial genomic DNA was extracted from intestinal samples of juvenile Asian seabass. The study included five coccidia uninfected and seven coccidia infected samples. DNA extraction was performed using the PureLink Genomic DNA Mini Kit (Invitrogen, MA, USA), following the manufacturer’s protocol. In brief, intestinal samples were homogenized, and DNA was isolated by binding to a silica membrane, followed by elution in a low-salt buffer. The concentration and purity of the extracted DNA were assessed using a Nanodrop spectrophotometer (Thermo Fisher Scientific Inc., DE, USA). All DNA samples were stored at −20 °C until further processing. Library preparation and sequencing were outsourced to NovogeneAIT Genomics Singapore Pte. Ltd. (Singapore). For each sample, a DNA library was prepared using the Nextera DNA Flex Library Prep Kit (Illumina, CA, USA). Amplification of the bacterial 16S rRNA gene was targeted specifically to the V4 hypervariable region using primers 515F (5’-GTGCCAGCMGCCGCGGTAA-3’) and 806R (5’-GGACTACHVGGGTWTCTAAT-3’). Sequencing was performed using the NovaSeq 6000 Sequencing System (Illumina, Inc., CA, USA), generating 253 bp paired-end reads with the NovaSeq 6000 Reagent Kits v1.5. The raw sequencing data generated from this study is available under BioProject ID: PRJNA1156012 at GenBank (https://www.ncbi.nlm.nih.gov/bioproject/PRJNA1156012).

### Microbiota analysis

Microbial sequence data were processed using the Quantitative Insights Into Microbial Ecology 2 (QIIME2) pipeline, version 2022. [22]. Paired-end sequence reads were initially demultiplexed, quality-filtered, and denoised using the DADA2 plugin within QIIME2 to correct sequencing errors, remove chimeric sequences, and merge paired-end reads. The resulting amplicon sequence variants (ASVs) were used for downstream analysis. Sequencing yielded a total of 1,302,868 high-quality reads (mean ± SD: 108,572 ± 30,930; median: 114,863; range: 52,556–150,594 reads per sample). Rarefaction analyses were performed at the minimum sequencing depth of 52,556 reads, and Good’s coverage was calculated for each sample at this depth (Supplementary Table S1; Supplementary Fig. S1). Taxonomic assignment was performed using the Silva 138 99% taxonomy classifier [23, 24], with sequences aligned via the mafft plugin in QIIME2 to generate phylogenetic relationships. To assess microbial diversity, both alpha and beta diversity metrics were calculated. For alpha diversity, measures of observed ASVs, Shannon diversity index, and Pielou’s evenness were computed to evaluate the richness and evenness of microbial communities within each sample. Beta diversity was assessed using the Bray–Curtis dissimilarity matrix to capture differences in microbial community composition between the coccidia infected and coccidia uninfected groups. The beta diversity results were visualized through Principal Coordinate Analysis (PCoA). Differential abundance analysis was conducted using Linear Discriminant Analysis (LDA) effect size (LEfSe) to identify significantly different bacterial taxa between the infected and uninfected groups [25]. Relative abundances of the identified taxa were further compared between the groups.

### Semi-quantitative intestinal inflammation

Intestinal tissues were individually fixed in 10% buffered formalin for 24 h, followed by standard histological processing and paraffin embedding [26]. Sections of 4 μm thickness were mounted on glass slides and stained with hematoxylin and eosin (H&E) for histopathological evaluation. Inflammatory changes were assessed in three key areas: submucosal inflammation, intraepithelial lymphocyte infiltration, and overall extent of inflammation. Cell identification followed the criteria described by Clauss et al. [27]. Lesion grading was conducted using a semi-quantitative scoring system adapted from Suyapoh et al. [1]. For each intestinal section, five non-overlapping microscopic fields within the mucosal and submucosal layers were examined under 40 × magnification. The severity of lesions was scored on a scale of 0–3, corresponding to: 0 = absent, 1 = mild, 2 = moderate, and 3 = severe. The grading was based on the proportion of affected area relative to unaffected tissue, with the following criteria: absent = no lesion or ≤ 1% involvement; mild = 2–25%; moderate = 26–50%; and severe = > 50%. The assessment was performed in a blinded manner, and the mean score from the five fields was calculated to represent the severity of lesions for each fish.

### Statistical analysis

Statistical significance of microbial diversity and abundance differences was evaluated using the Kruskal–Wallis test and visualizations were generated using the microeco R package version 1.9.1 [28]. Morphometric parameters (width, length, and weight) between infected and uninfected groups were compared using the Mann–Whitney U test (two-sided). A *p*-value < 0.05 was considered statistically significant.

## Results

### Anamnesis, clinical examination, and external parasitological assessment

Juvenile Asian seabass (*Lates calcarifer*) originating from a marine cage farm in Satun Province, Thailand, were reared in an eastern pond located adjacent to a natural water source, where a sieve system was installed to prevent the entry of wild fish, and throughout the rearing period no chemical treatments were applied to the pond, while the diet provided consisted of commercial fish pellets supplemented with minced fresh fish. The health status of the 13 sampled fish was evaluated by means of both premortem and postmortem examination, and morphometric measurements revealed an average body width of 2.83 ± 0.83 cm, a total length of 8.74 ± 2.08 cm, and a body weight of 15.13 ± 10.17 g.

Clinically, several individuals displayed slightly darker skin pigmentation and signs of emaciation, whereas others were observed swimming near the water surface with rapid opercular movements, which are suggestive of respiratory compromise. External parasitological assessment demonstrated the presence of infestations by *Trichodina* spp. and gill flukes, with all examined fish being infected to varying degrees of severity, and the mean infestation grade was 0.80 ± 1.01 for Trichodina and 0.13 ± 0.52 for gill flukes, which corresponded overall to a light ( +) level of infection.

Postmortem findings in affected fish included fin erosions, detached scales, and multifocal areas of skin erosion, together with small focal hemorrhages on the ventral surface. In addition, most affected individuals exhibited swollen and hyperemic gill filaments, consistent with parasitic irritation and secondary vascular compromise. Internal examination revealed a pale and enlarged liver, a distended gall bladder, and occasional intestinal segments that appeared diffusely reddened, suggestive of congestion or localized hemorrhage. In contrast, uninfected fish were clinically normal and demonstrated no evidence of external or internal lesions upon examination.

### Coccidia detection

Coccidia detection by morphological examination identified developmental stages at three epithelial sites—epicellular, intracytoplasmic, and extracellular (subepithelial) layers—with parasites predominantly located in the extracellular or subepithelial positions across all intestinal regions. Quantitative assessment of parasite burden demonstrated that the positive group exhibited an infection intensity of 398.95 ± 234.05, compared with 0.00 ± 0.00 in the negative group (*p* = 0.0013), thereby confirming a markedly higher parasitic load in infected fish.

### Comparison of health parameters between groups

Morphometric analysis revealed that coccidia-infected juvenile Asian seabass (positive group) had an average body width of 2.91 ± 0.75 cm, total length of 9.21 ± 2.15 cm, and body weight of 16.44 ± 9.95 g, whereas uninfected fish (negative group) exhibited an average body width of 2.00 ± 0.00 cm, total length of 8.20 ± 2.10 cm, and body weight of 14.20 ± 11.71 g. Statistical comparison indicated that body width was significantly greater in the positive group (*p* = 0.015), while differences in total length (*p* = 0.157) and body weight (*p* = 1.000) were not significant. In terms of external parasitic infestations, the positive group exhibited a mean grading score of 0.56 ± 1.01, whereas the negative group had a higher mean score of 1.60 ± 0.89, though this difference was not statistically significant (*p* = 0.102). Gill fluke infestation was generally rare, with mean scores of 0.00 ± 0.00 in the positive group and 0.40 ± 0.89 in the negative group.

Clinically, signs were more apparent in the positive group, with infected fish frequently presenting with darker skin pigmentation, emaciation, and abnormal swimming behavior, including surface orientation and rapid opercular movements, whereas fish in the negative group appeared clinically normal. Gross pathological examination further demonstrated that fish in the positive group often displayed fin erosions, scale detachment, and cutaneous hemorrhages, along with swollen and hyperemic gill filaments; internal lesions included pale, enlarged livers, distended gall bladders, and intestinal segments with diffuse congestion or hemorrhage. In contrast, fish in the negative group showed no remarkable external or internal pathological alterations.

### Piscine intestinal coccidia infection increases gut microbial diversity in juvenile Asian seabass

**Fig. 1:**
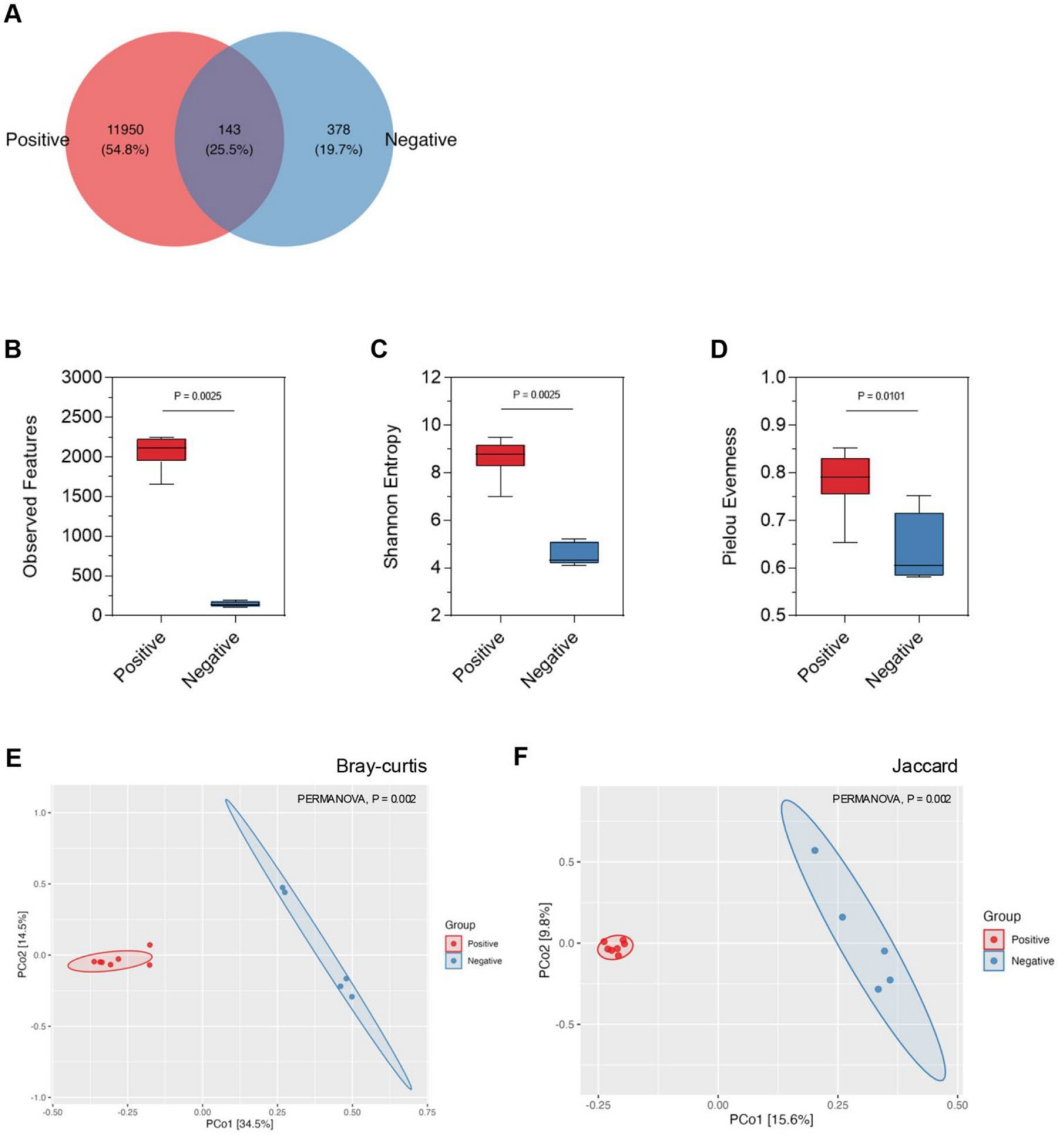
Composition and diversity of juvenile Asian seabass gut microbiota. **A** The Venn diagram shows Unique and shared ASVs between the coccidia-positive and negative groups. **B** The alpha diversity of both groups was analyzed using observed features, (**C**) Shannon entropy, and (**D**) Pielou’s evenness. Means were compared using the Kruskal–Wallis test (*P* < 0.05). The beta diversity between the two groups was calculated and visualized by Principal Coordinates Analysis (PCoA) based on (**E**) Bray–Curtis and (**F**) Jaccard indices. Permutational multivariate analysis of variance (PERMANOVA) were tested for statistical significance (*P* < 0.05)

To investigate the impact of piscine intestinal coccidia infection on gut microbiota, we first examined the differences in microbial diversity between the gut microbiota of piscine intestinal coccidia infected and uninfected Asian seabass. DNA sequencing and grouping of amplicon sequence variants (ASVs) were used to quantify microbial diversity in the gut content of both infected and uninfected groups. The total high-quality DNA sequences were obtained from the gut microbiota of juvenile Asian seabass and subsequently denoised into 12,614 distinct ASVs. The fish infected with piscine intestinal coccidia, referred to as the positive group, exhibited a markedly higher number of ASVs (12,093 ASVs) compared to the uninfected group (negative group), which had only 521 ASVs. This indicates a more complex and diverse microbial ecosystem in the gut of coccidia-infected fish. Despite the large difference in ASV numbers, only 143 ASVs were shared between the two groups, reflecting a minimal overlap in microbial composition. The positive group had 11,950 unique ASVs, while the negative group harbored only 378 unique ASVs (Fig. 1A), suggesting that coccidia infection introduces a significant number of novel microbial species or strains that are not present in healthy fish.

### Piscine intestinal coccidia infection changes the variety and composition of microbial taxa in the gut microbiota of juvenile Asian seabass

The changes in gut microbial diversity led us to further investigate species variety through richness and evenness sing alpha diversity analysis, as well as to examine differences in microbial composition through beta diversity in piscine intestinal coccidia-infected and uninfected juvenile Asian seabass. The alpha bacterial diversity was significantly higher in the infected group compared to the uninfected group. This was confirmed through multiple diversity metrics, including observed features, the Shannon diversity index, and Pielou’s evenness. Specifically, the positive group demonstrated greater microbial richness, meaning a higher number of distinct microbial taxa were present as well as higher evenness (Fig. 1B-D). In contrast, the negative group showed reduced richness and evenness, consistent with a more simplified and less microbial community in the absence of coccidia infection.

Beta diversity, which examines the differences in microbial composition between the two groups, further highlighted the substantial impact of coccidia infection on gut microbiota. Principal Coordinates Analysis (PCoA) based on both Bray–Curtis dissimilarity and Jaccard index revealed a clear and distinct separation between the positive and negative groups, underscoring the dissimilar microbial structures between infected and uninfected fish (Fig. 1E-F). This separation indicates that the overall community structure in the positive group had shifted significantly compared to the negative group, with coccidia infection driving changes in both the presence and abundance of specific microbial taxa.

### Piscine intestinal coccidia infection alters gut microbiota composition in juvenile Asian seabass at multiple taxonomic levels

**Fig. 2:**
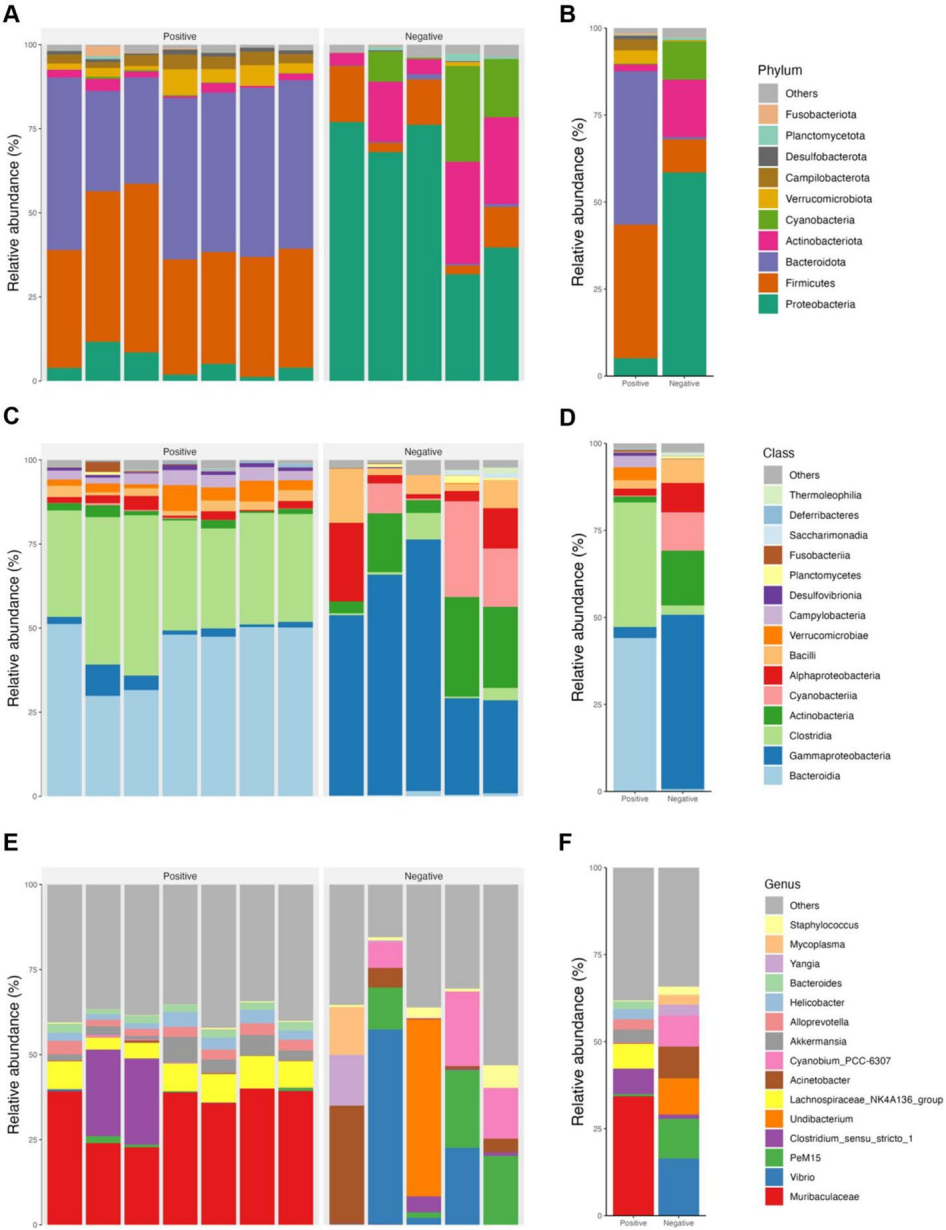
Taxonomic profile of juvenile Asian seabass gut microbiota. The relative abundances of microbial phyla in coccidia-positive and negative groups are shown by (**A**) individual sample and (**B**) averaged by group. Similarly, the relative abundances of microbial classes and genus are displayed (**C**, **E**) for each sample and (**D**, **F**) group

The impact of piscine intestinal coccidia on the changes in the taxonomic composition of gut microbiota between the infected and uninfected groups were further examined. Taxonomic classification of the gut microbiota revealed distinct differences in the dominant microbial groups between the coccidia-infected (positive) and uninfected (negative) fish. At the phylum level, the gut microbiota of the positive group was primarily composed of Bacteroidota (44.10% ± 9.24) and Firmicutes (38.37% ± 6.47), which together accounted for more than 82% of the total microbial community (Fig. 2A). In contrast, the negative group’s gut microbiota was dominated by Proteobacteria (58.50% ± 21.32), with smaller contributions from Actinobacteriota and Cyanobacteria (Fig. 2B). A comparative analysis of the relative abundances of major phyla between the two groups showed that Proteobacteria and Actinobacteriota were significantly less abundant in the positive group, suggesting that coccidia infection suppresses these phyla. Conversely, Bacteroidota, Firmicutes, Campilobacteriota, and Verrucomicrobiota were significantly more abundant in the positive group (Fig. 2E). At the class level, the differences in microbial composition were even more pronounced. In the positive group, the most dominant classes were Bacteroidia (44.08% ± 8.56) and Clostridia (35.83% ± 6.44), while the negative group was dominated by Gammaproteobacteria (50.06% ± 19.11) (Fig. 2C – D). At the genus level, the most dominant of gut microbiota in positive group was Muribaculaceae (33.75% ± 7.07), while the most abundance in negative group was Vibrio (21.08% ± 19.00) followed by PeM15, Undibacterium, Acinetobacter, and Cyanobium_PCC-6307 (Fig. 2E – F). The shift in microbial classes indicates that coccidia infection selectively favors certain bacterial taxa while suppressing others, resulting in a dramatically altered gut microbial landscape.

### Identification of microbial biomarkers

**Fig. 3:**
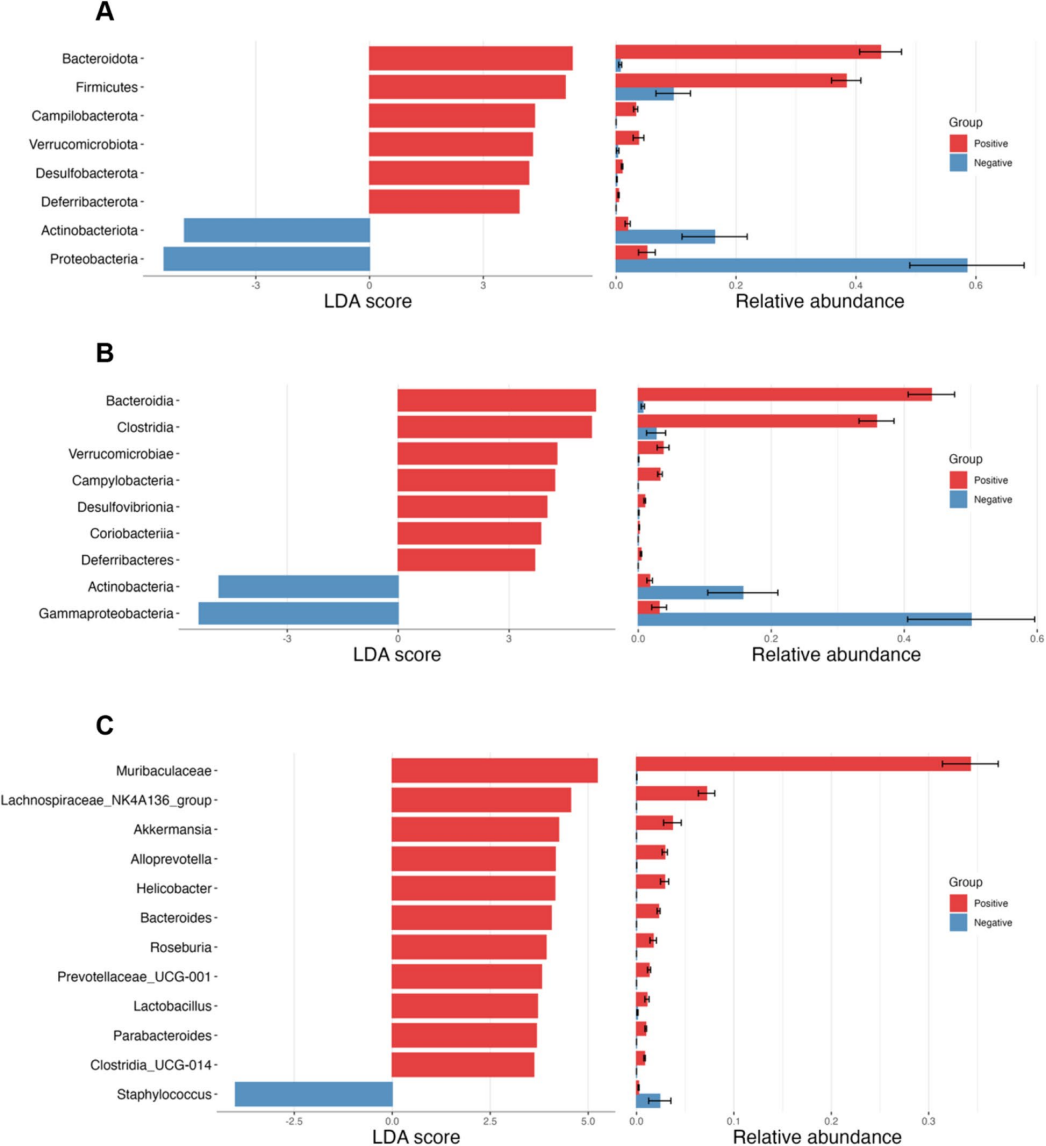
Differential abundance analysis of juvenile Asian seabass gut microbiota between coccidia-positive and negative groups. Linear Discriminant Analysis (LDA) scores were calculated to identify microbial biomarkers and the relative abundance of significant taxa were compared in both groups and showed at the (**A**) phylum, (**B**) Class, and (**C**) Genus level

To identify variations in microbial taxa abundance between piscine intestinal coccidia-infected and uninfected juvenile Asian seabass, we conducted differential abundance analysis using the linear discriminant analysis (LDA) effect size (LEfSe) method. This allowed us to determine which microbiota were significantly more or less abundant in each group. In coccidia-infected group, the abundances of the phyla Proteobacteria and Actinobacteriota were lower than observed in healthy fish, while the abundances of the phyla Bacteroidota, Firmicutes, Campilobacterota, Verrucomicrobiota, Desulfobacterota, and Deferribacterota were higher (Fig. 3A). At the class level, Gammaproteobacteria and Actinobacteria presented decreased abundance in coccidia-infected fish compared to the healthy fish. In contrast the class Bacteriodia, Clostridiam, Verrucomicrobiae, Campylobacteria, Desulfovibrioniam Coriobacteriia, and Deferribacteres in infected fish were found to be more abundant than in healthy fish (Fig. 3B). At the genus level, Muribaculaceae, Lachnospiraceae_NK4A136_group, Akkermansia, Alloprevotella, Helicobacter, Bacteroides, Roseburia, Prevotellaceae_UCG-001, Lactobacillus, Parabacteroides, and Clostridia_UCG-014 were identified as key biomarkers (Fig. 3C). However, in coccidia-infected fish, the abundance of Staphylococcus was decreased compared to healthy. These findings further emphasize the significant shifts in microbial composition caused by coccidia infection and highlight potential microbial markers that could be useful for diagnosing or studying the impact of intestinal coccidia in fish.

### Piscine intestinal coccidia infection increases pathogenic genera in juvenile Asian seabass

**Fig. 4:**
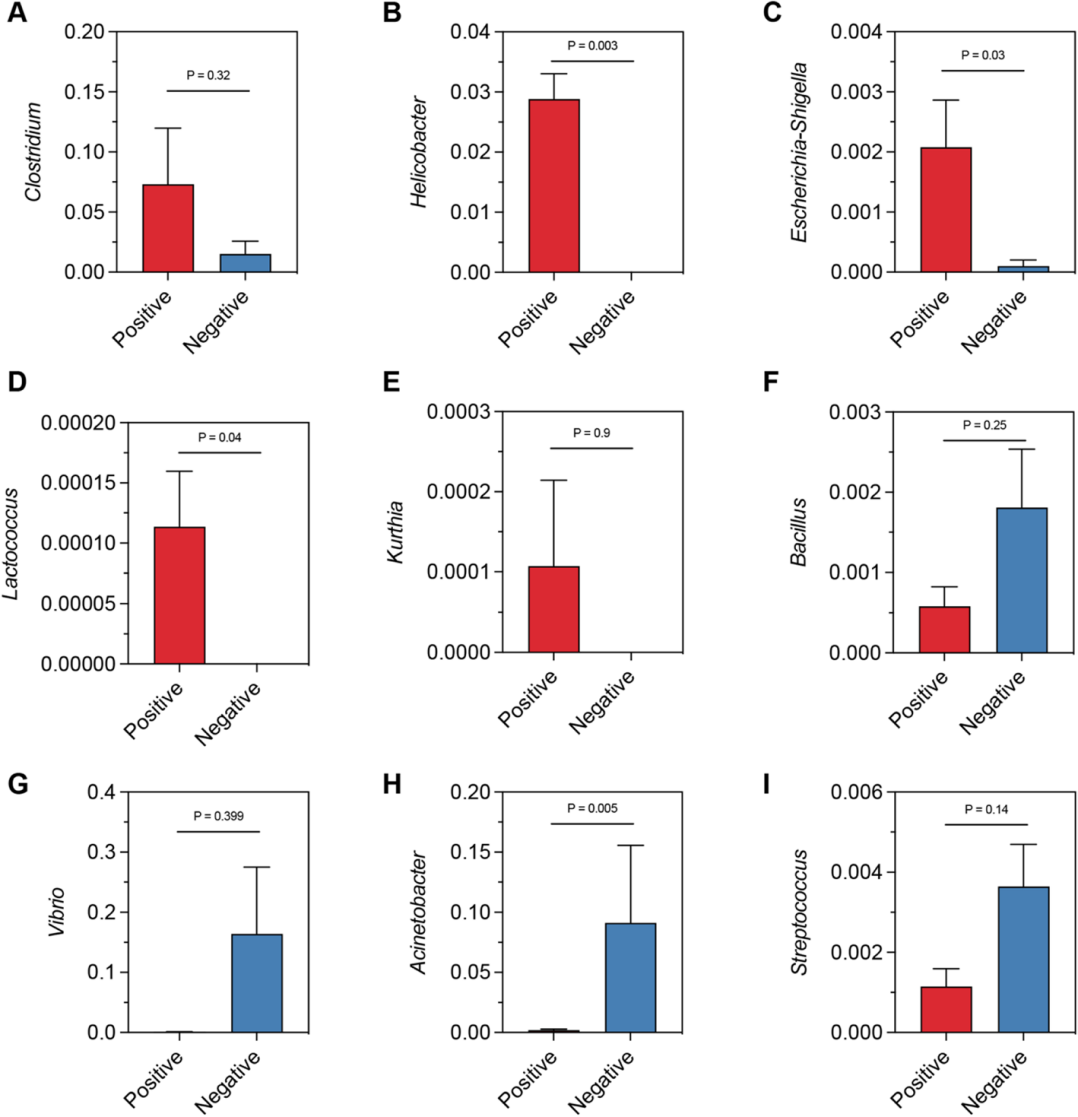
Relative abundance of important genera in Juvenile Asian seabass gut microbiota. The relative abundances of microbial pathobionts, including (**A**) Clostridium, (**B**) Helicobacter, (**C**) Escherichia-Shigella, (**D**) Lactococcus, and (**E**) Kurthia, were high in the coccidia-positive group. While, the relative abundances of (**F**) Bacillus, (**G**) Vibrio, (**H**) Acinetobacter, and (**I**) Streptococcus were significantly high in the negative group. Means were compared using the Kruskal–Wallis test (*P* < 0.05)

Frequent pathogenic bacteria was further analyzed in both fish groups. The positive group had significantly higher relative abundances of several genera associated with pathogenicity, including *Clostridium*, *Helicobacter*, *Escherichia-Shigella*, *Lactococcus*, and *Kurthia* (Fig. 4A-E). These genera are known for their ability to exploit disruptions in gut homeostasis, which may contribute to disease progression in coccidia-infected fish. In contrast, the negative group exhibited higher relative abundances of genera such as *Bacillus*, *Vibrio*, Acinetobacter, and *Streptococcus*, which are commonly associated with environmental bacteria or opportunistic pathogens (Fig. 4F-I).

### Piscine intestinal coccidia infection disrupts gut microbial balance and causes gut inflammation in juvenile Asian seabass

**Fig. 5:**
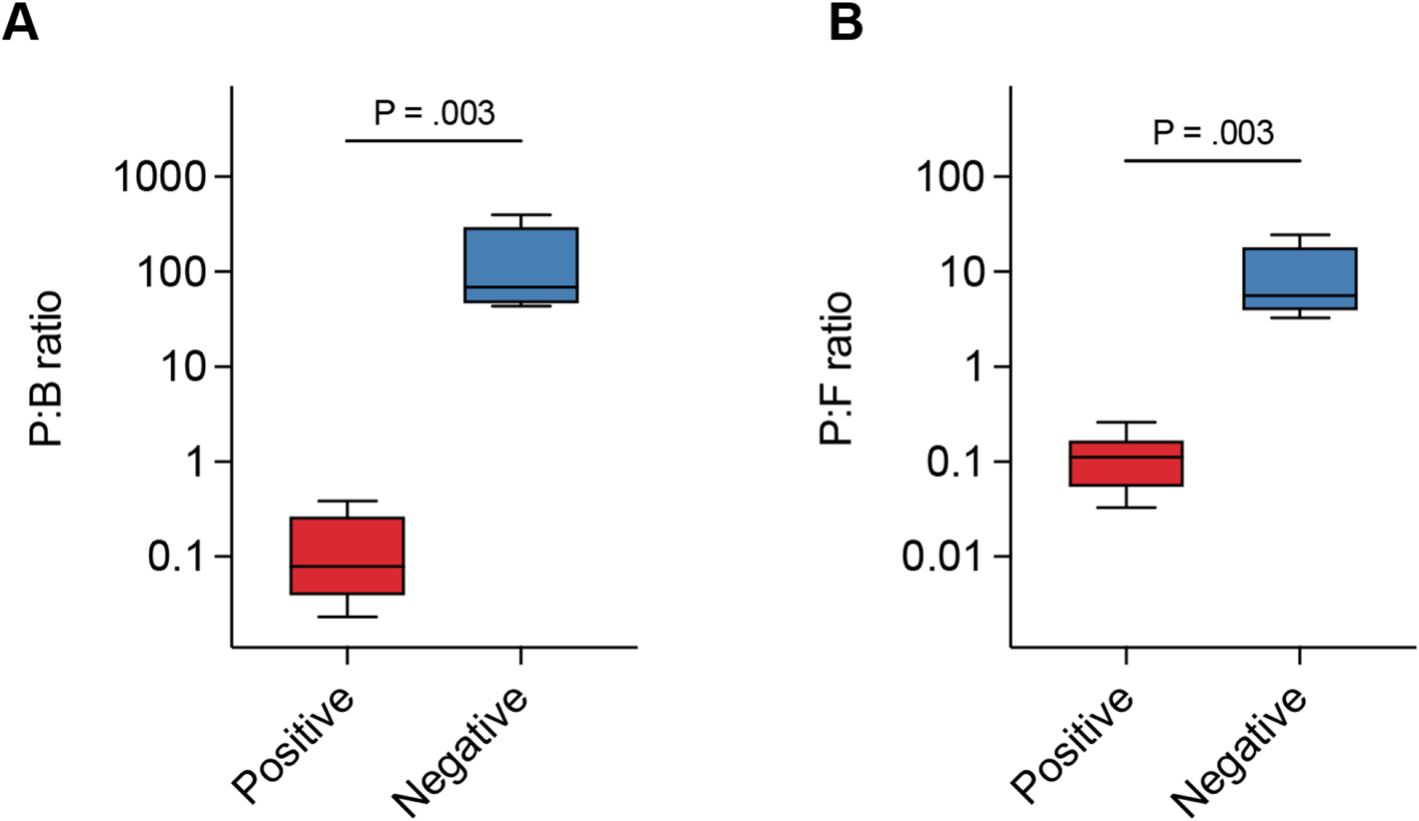
The key indicators of gut health in juvenile Asian seabass gut microbiota. The ratio of (**A**) Proteobacteria to Bacteroidota (P:B ratio) and (**B**) Proteobacteria to Firmicutes (P:F ratio) was calculated in the coccidia-positive group compared to the negative group. Means were compared using the Kruskal–Wallis test (*P* < 0.05)

Based on the hypothesis that piscine intestinal coccidia infection alters the gut microbiome and promotes the proliferation of pathogenic bacteria, we further investigated the potential role of coccidia in disrupting the typical balance of gut bacteria. We assessed the ratios of Proteobacteria to Bacteroidota (P:B ratio) and Proteobacteria to Firmicutes (P: F ratio) and compared the relative abundances of pathogenic genera between coccidia-infected and uninfected groups. Overalls, both ratios were significantly reduced in the positive group compared to the negative group, indicating a profound alteration in microbial equilibrium (Fig. 5). The lower P:B and P:F ratios in the positive group suggest that coccidia infection disrupts the typical balance of gut bacteria, reducing the prevalence of Proteobacteria and favoring the expansion of Bacteroidota and Firmicutes.

**Fig. 6:**
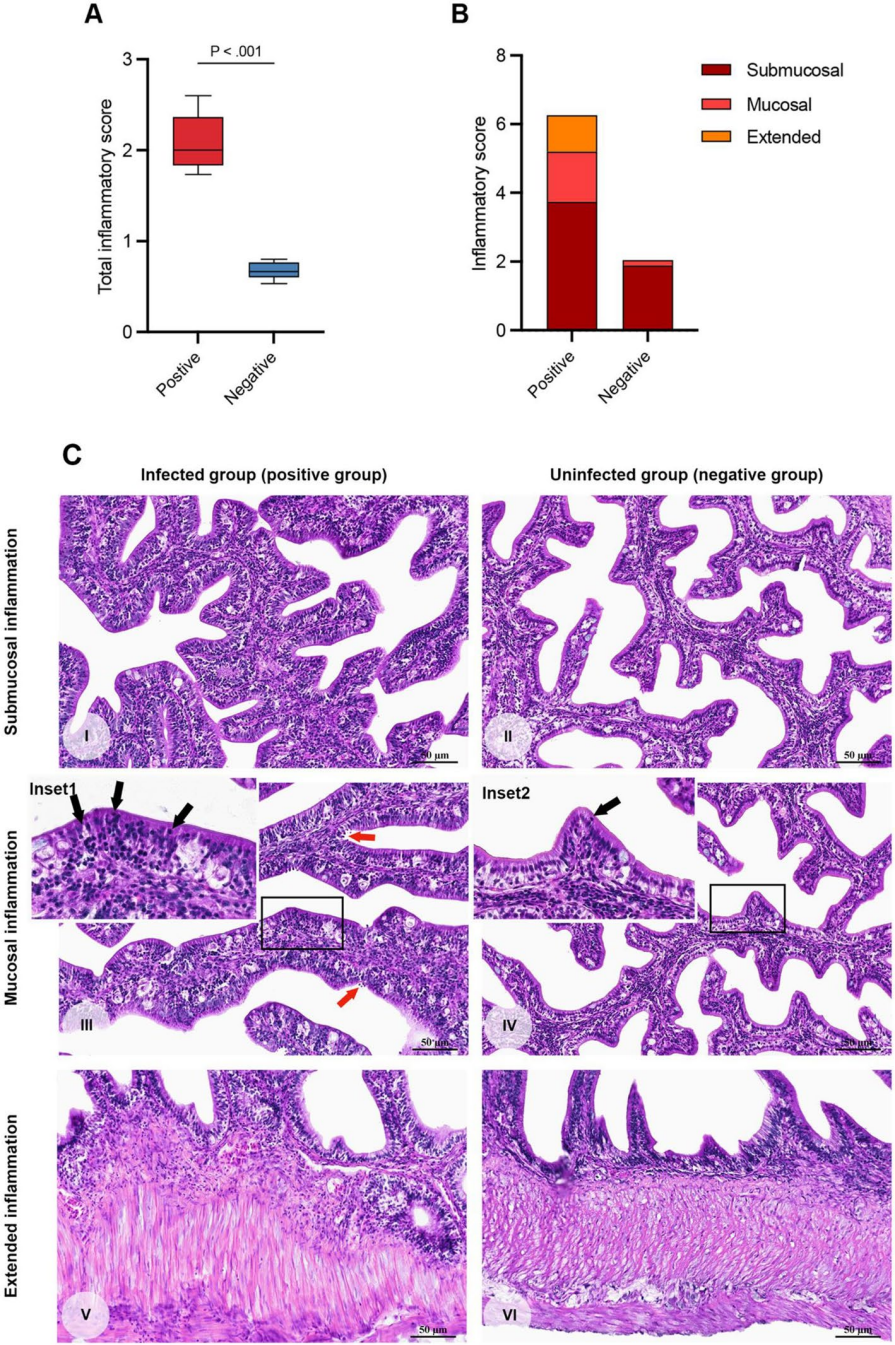
A comparative analysis of inflammation and histopathological lesions in the intestinal tissues of coccidia-infected and non-infected juvenile Asian seabass. Histopathological changes in inflammatory lesions: (I-II) Submucosal inflammation; (III-IV) Submucosal or intraepithelial lymphocyte infiltration (black arrow; insets 1–2). Increased lymphocyte infiltration was observed in the positive group, with coccidia present in the supraepithelial area (III, red arrow). (V-VI) Extension of inflammation. These inflammatory patterns were predominantly observed in the coccidia-infected group (I, III, V), while only a low-grade inflammatory response was detected in the non-infected group (II, IV, VI). (I-VI = H&E, original magnification × 40, scale bar = 50 µm)

Evidence of the impact of gut microbiome alteration induced by coccidiosis was assessed through histopathological examination. Histopathological analysis demonstrated significantly increased inflammatory cell infiltration in coccidia-infected fish (positive group) compared to non-infected individuals (negative group) (Fig. 6A). In the positive group, all three types of inflammation—submucosal, mucosal, and extended (transmural)—were observed, whereas in the negative group, only submucosal and mucosal inflammation were present, with lower severity grades (Fig. 6B). Histologically, inflammation was most prominent in the submucosal layer (Fig. 6CI–CII), followed by the mucosal and intraepithelial regions (Fig. 6CIII–CIV), and in more severe cases, extended into the muscularis layer (Fig. 6CV–CVI). The infiltrating leukocyte population was predominantly composed of mononuclear cells, including lymphocytes and macrophages, with granulocytes such as eosinophils and neutrophils occurring less frequently. These cells primarily accumulated in the lamina propria of the villi, leading to notable papillary distension—a hallmark more frequently observed in the positive group (Fig. 6CI, CIII, CV). Intraepithelial lymphocyte infiltration was also identified, characterized by lymphocyte migration into and residence within the cytoplasm of enterocytes, a feature commonly associated with coccidia infection and predominantly observed in infected fish. In some cases, the inflammatory response extended beyond the muscularis into the full thickness of the intestinal wall, reflecting a more advanced stage of inflammation.

## Discussion

Piscine intestinal coccidia are common parasites found in fish populations and can lead to symptomatic illnesses and even mortality [29]. In Asian seabass, the most frequently reported genera are *Eimeria* spp. and *Cryptosporidium* spp. [3]. Based on morphological characteristics, the present study also identified intestinal coccidia consistent with the genus *Eimeria*. Infections with these protozoa are known to elicit chronic inflammatory responses that contribute to intestinal pathology [1, 2, 6], and such inflammation is often associated with alterations in the gut microbiota [30]. Our findings provide new insights into the impact of piscine intestinal coccidia on the gut environment, suggesting that these parasites may create an “open niche” that facilitates colonization by diverse microbial taxa, including potentially pathogenic and zoonotic genera, as demonstrated in juvenile Asian seabass. Infected fish exhibited markedly greater microbial richness and diversity—approximately four times higher than that observed in uninfected fish—a trend consistent with reports of coccidiosis in higher vertebrates [31, 32]. Nevertheless, the inability to identify coccidia to genus or species level in this study limits direct associations between specific taxa and microbial alterations. Future research employing molecular or ultrastructural methods will be essential to achieve species-level resolution and to clarify how different coccidian species modulate gut microbial dynamics.

Change in the ratio of pivotal phyla (P:F or P:B) in our study indicated a decreasing ratio in the piscine intestinal coccidia-infected group, reflecting signatures of fish intestinal alteration and inflammation [33]. This pattern has also been observed in various fish species, such as grouper (*Epinephelus coioides*) [34], gibel carp (*Carassius gibelio*) [35], and Atlantic salmon (*Salmo salar*) [36]. Analysis of the microbiome at the phylum level reveals notable changes associated with piscine intestinal coccidia infection, characterized by an increase in the abundance of Bacteroidota and Firmicutes. Similarly, in fish infected with the parasite *Pomphorhynchus* sp., there is an increase in Firmicutes and Fusobacteriota, along with notably lower proportions of Actinobacteriota and Alphaproteobacteria [37]. In contrast, the gut microbiome of coccidia uninfected fish is predominantly composed of Proteobacteria, Actinobacteriota, and Cyanobacteria. The prevalence of Proteobacteria in the healthy gut microbiome of uninfected fish is consistent with previous studies on normal juvenile stage of Asian seabass, which also identified Proteobacteria as the dominant phylum [38, 39]. On the other hand, there’s been a different take on how microbial changes occur with various parasitic infections, like *Tetracapsuloides bryosalmonae*, *Khawia japonensis*, *Atractolytocestus tenuicollis*, and certain flukes in different fish species [40–42]. Likewise, different bacterial diseases, such as Tenacibaculosis, Furunculosis, Streptococcosis, and Aeromoniasis, have shown their own unique shifts in microbial communities [43–46]. We propose that variations in pathogens, fish species, developmental stages, geographic distribution, and environmental factors may contribute to these differing patterns.

At the genus level, LDA analysis reveals an increase in *Muribaculaceae* (phylum Bacteroidota), *Prevotellaceae*, *Lachnospiraceae*_NK4A136, *Oscillospirales* (phylum Firmicutes), and *Akkermansia* (phylum Verrucomicrobiota) in the juvenile Asian seabass gut microbiome, indicating their potential as biomarkers for exposure to piscine intestinal coccidia. A similar trend has been observed in poultry coccidiosis, where an increase in Firmicutes, Bacteroidaceae, and Oscillospirales, as well as Bacteroidota, was noted at 4 days post-infection in the cecum and at later stages in the jejunum [32]. In contrast, research on rabbit coccidiosis has demonstrated an increase in Proteobacteria, Enterobacteriales, Enterobacteriaceae, Gammaproteobacteria, and Escherichia [47]. In Hu lambs with coccidiosis, there is an increase in Clostridialcs, Clostridiaceae, *Clostridium-*sensu*-stricto*−1, Peptostreptococcale-Tissierellales, Porphyromonadaccae, Porphyromonas, Fusobactcriota, Fusobacteriia, Fusobacteriales, Fusobacteriaceae, *Fusobacterium*, Proteobacteria, Gammaproteobacteria, Enterobacteriaceae, Escherichia-Shigella, Bacteroidaceae, Bacteroides, Oscillospiraceae [48]. These differing patterns of microbiota changes among fish, poultry, and mammals may reflect the evolutionary signatures of their hosts, suggesting that ecological and evolutionary forces influence both the host and its resident microbiome [49].

Our study demonstrates the potential impact of piscine intestinal coccidiosis on the overgrowth of microbial genera with pathogenic potential in the gut of juvenile Asian seabass. Infected fish showed significantly higher abundances of *Clostridium*, *Lactococcus*, and *Kurthia*, genera known to exploit disruptions in gut homeostasis [50], thereby contributing to disease progression. Additionally, zoonotic taxa such as *Helicobacter* and *Escherichia–Shigella*, although primarily recognized as human pathogens, were also detected. Previous studies have reported *Shigella* colonization in fish [51] and identified tilapia as potential reservoirs of *Helicobacter pylori*, raising the possibility that these bacteria may act as emerging fish-borne pathogens [52, 53]. In contrast, the uninfected fish displayed higher relative abundances of genera such as *Bacillus*, *Vibrio*, *Acinetobacter*, and *Streptococcus.* While these groups contain recognized fish pathogens [54, 55], many members function as environmental or opportunistic taxa, and several *Bacillus* species are widely utilized as probiotics in aquaculture due to their immunomodulatory and antimicrobial benefits [56]. Because our 16S rRNA sequencing data were limited to the genus level, functional roles could not be assigned with certainty, highlighting the need for future studies with species- or strain-level resolution to better define their contributions.

Although the sample size is limited and restricts generalization, this exploratory study is the first to demonstrate an association between coccidial infection and gut microbiome alterations in juvenile Asian seabass. All fish were obtained from a single facility under uniform rearing conditions, thereby minimizing potential confounding factors. Histopathological examination revealed increased intestinal inflammation in infected fish, supporting a link between parasite-induced epithelial disruption, the creation of open ecological niches within the gut, and colonization by opportunistic bacteria. This observation is consistent with reports of coccidiosis in Asian seabass and other marine fish [1, 3, 18, 26]. The pathogenic process arises as coccidia multiply within intestinal epithelial cells, causing cell rupture and mucosal damage, which creates conditions favorable for bacterial proliferation [57]. Such epithelial injury also facilitates bacterial penetration into the mucosa, frequently leading to severe inflammation and focal necrosis [58]. The observed increase in microbial diversity in infected fish may therefore reflect an “open niche” phenomenon, as indicated by the expansion of multiple bacterial genera. These findings support the hypothesis that alterations in gut integrity and microbial niches may exacerbate intestinal pathology. However, larger-scale studies are needed to validate these results and to further clarify the role of gut microbiome changes in the pathogenesis of piscine intestinal coccidiosis.

Interestingly, recent studies suggested that piscine intestinal coccidiosis in fish frequently links to systemic secondary infections from both pathogenic and opportunistic bacteria, many of which are typically part of the fish's gut flora [6, 59]. The mechanisms by which piscine intestinal coccidiosis modifies the gut microbiome remain largely unexplored. However, insights from other vertebrate coccidiosis indicate that both direct and indirect interactions may play a role [60]. Direct interactions involve relationships between the parasite and bacteria that can influence one another, although clear evidence supporting this mechanism is currently lacking [60]. Indirect interactions resulting from coccidia infection include mechanical injury, which can impair tight junctions and lead to apoptosis of intestinal epithelial cells [61]. This disruption can modify the intestinal chemical barrier, including components such as mucin (MUC), antimicrobial peptides (AMPs), and other factors [62], thereby creating conditions conducive to the growth of conditionally pathogenic bacteria [63]. In the pathogenesis of piscine intestinal coccidiosis, the parasite induces mechanical injury and modulates inflammatory responses, which can result in the production of free radicals, DNA damage, and apoptosis [1, 6, 26]. These processes collectively contribute to alterations in the gut microbiota.

## Conclusions

In conclusion, our study demonstrates that piscine intestinal coccidiosis significantly alters the intestinal microbiota of juvenile Asian seabass, resulting in open niche of microbiome in gut. Several bacterial genera showed increased relative abundance in infected fish, some of which include species known as potential pathogens, while others may also comprise commensal or opportunistic members. Given that our sequencing data were limited to the genus level, these findings should be interpreted with caution. Future research incorporating species- or strain-level approaches is needed to clarify the functional roles of these taxa, as well as to investigate the influence of environmental factors, developmental stages, and host species. Such studies will advance our understanding of host–parasite–microbiota interactions and contribute to the development of strategies to improve fish health and mitigate the impact of coccidia infections.

## Supplementary Information

Supplementary Material 1.

## Data Availability

No datasets were generated or analysed during the current study.

## Abbreviations

AMPs: Antimicrobial peptides
ASVs: Amplicon sequence variants
DNA: Deoxyribonucleic acid
LDA: Linear discriminant analysis
LEfSe: Linear discriminant analysis effect size
MUC: Mucin or mucus-glycoprotein
P:B ratio: Proteobacteria-to-bacteroidota ratio
P:F ratio: Proteobacteria-to-firmicutes ratio
PCoA: Principal coordinate analysis
PCR: Polymerase chain reaction
QIIME2: Quantitative insights into microbial ecology 2
16S rRNA gene: 16S ribosomal ribonucleic acid gene
