# Supplementary material for: Pathogenic piscine intestinal coccidia infection alters gut microbiome in juvenile Asian seabass (Lates calcarifer)
Source: BMC Vet Res. 2025 Dec 18;22:57. doi: 10.1186/s12917-025-05168-y (PMC12849306; doi:10.1186/s12917-025-05168-y)
Supplement: Supplementary file 1 — Supplementary Material 1. [file 12917_2025_5168_MOESM1_ESM.docx]

**Supplementary Data**

**Supplementary Table S1.** Summary of sequencing depth and Good’s coverage per sample after rarefaction to 52,556 reads.

| **Sample ID** | **Frequency** | **Good’s coverage  (52,556 reads)** |
| --- | --- | --- |
| SM18 | 150,594 | 1.00 |
| SM22 | 141,920 | 1.00 |
| SM29 | 140,836 | 1.00 |
| SC09 | 129,764 | 1.00 |
| SP09 | 118,207 | 1.00 |
| SM43 | 117,511 | 1.00 |
| SP37 | 112,215 | 1.00 |
| SP22 | 104,399 | 1.00 |
| SC44 | 83,134 | 1.00 |
| SP06 | 79,573 | 1.00 |
| SM30 | 72,159 | 1.00 |
| SP26 | 52,556 | 1.00 |

Across all 12 samples, sequencing yielded a total of 1,302,868 high-quality reads (mean ± SD: 108,572 ± 30,930; median: 114,863; range: 52,556–150,594 reads per sample). Rarefaction curves for observed features, Shannon diversity, and Faith’s phylogenetic diversity approached plateaus for all samples (Supplementary Fig. S1), indicating sufficient sequencing depth. Good’s coverage at the rarefaction depth of 52,556 reads was 1.00 for all samples (Supplementary Table S1), confirming complete sampling coverage.

**
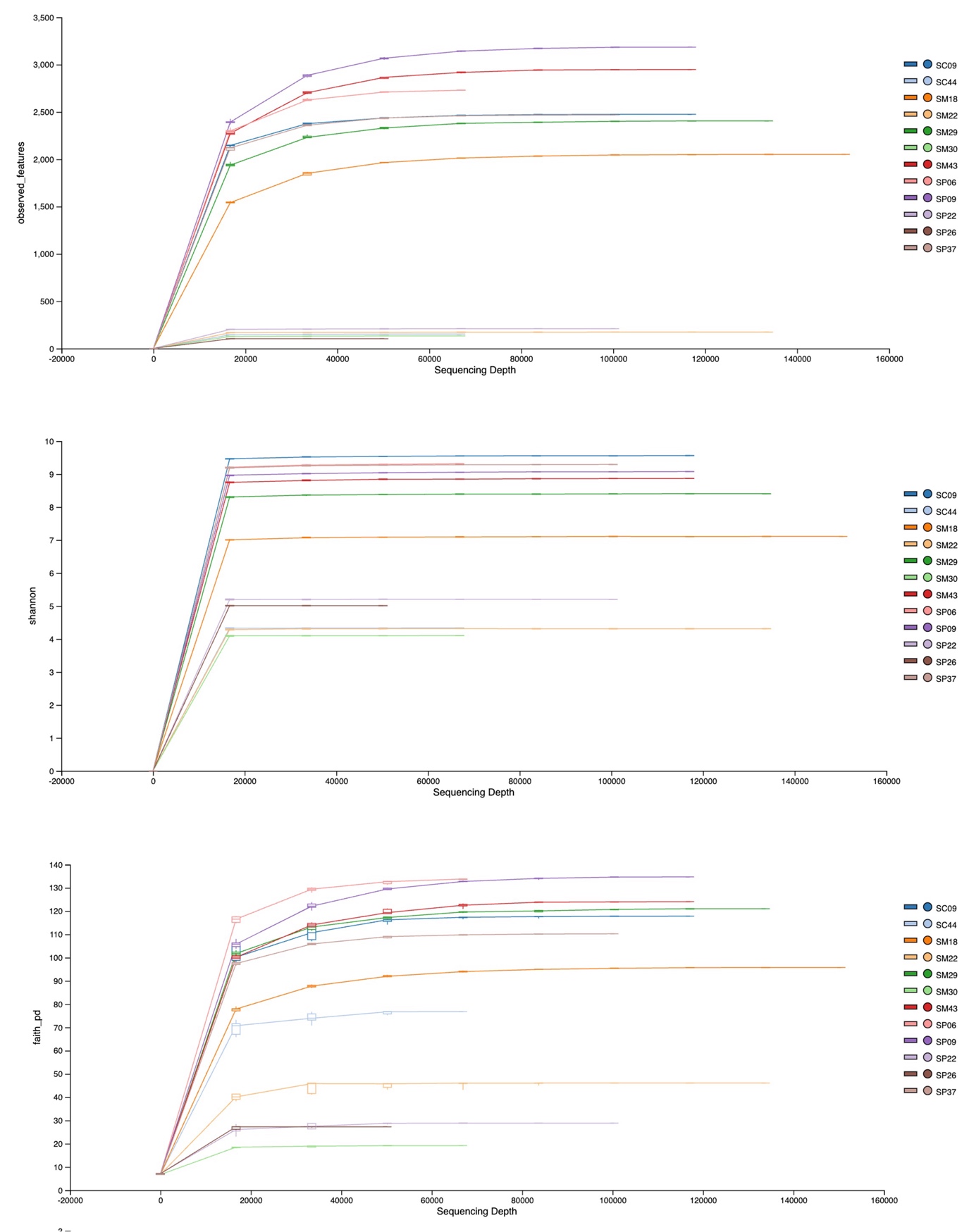
**

**Supplementary Figure S1.** Rarefaction curves for observed features, Shannon diversity, and Faith’s phylogenetic diversity for all 12 samples.
